# To Stop Bleeding

**Published:** 1882-11

**Authors:** 


TO STOP the flow of blood bind the cut with cobwebs and brown sugar, pressed on like lint; or, if you cannot procure these with fine dust of tea.

